# Raptor localization predicts prognosis and tamoxifen response in estrogen receptor-positive breast cancer

**DOI:** 10.1007/s10549-017-4508-x

**Published:** 2017-11-11

**Authors:** Josefine Bostner, Anya Alayev, Adi Y. Berman, Tommy Fornander, Bo Nordenskjöld, Marina K. Holz, Olle Stål

**Affiliations:** 1 Department of Clinical and Experimental Medicine, and Department of Oncology, Linköping University, Linköping, Sweden; 2 Department of Biology, Yeshiva University, New York, NY USA; 3 Department of Oncology, Karolinska University Hospital, Stockholm South General Hospital, Karolinska Institute, Stockholm, Sweden; 4 Department of Molecular Pharmacology, Albert Einstein College of Medicine, Bronx, NY USA; 5 Albert Einstein Cancer Center, Albert Einstein College of Medicine, Bronx, NY USA

**Keywords:** mTOR, Estrogen receptor (ER) α, Tamoxifen, Endocrine resistance, Luminal A

## Abstract

**Purpose:**

Deregulated PI3K/mTOR signals can promote the growth of breast cancer and contribute to endocrine treatment resistance. This report aims to investigate raptor and its intracellular localization to further understand its role in ER-positive breast cancer.

**Methods:**

Raptor protein expression was evaluated by immunohistochemistry in 756 primary breast tumors from postmenopausal patients randomized to tamoxifen or no tamoxifen. In vitro, the MCF7 breast cancer cell line and tamoxifen-resistant MCF7 cells were studied to track the raptor signaling changes upon resistance, and raptor localization in ERα-positive cell lines was compared with that in ERα-negative cell lines.

**Results:**

Raptor protein expression in the nucleus was high in ER/PgR-positive and HER2-negative tumors with low grade, features associated with the luminal A subtype. Presence of raptor in the nucleus was connected with ERα signaling, here shown by a coupled increase of ERα phosphorylation at S167 and S305 with accumulation of nuclear raptor. In addition, the expression of ERα-activated gene products correlated with nuclear raptor. Similarly, in vitro we observed raptor in the nucleus of ERα-positive, but not of ER-negative cells. Interestingly, raptor localized to the nucleus could still be seen in tamoxifen-resistant MCF7 cells. The clinical benefit from tamoxifen was inversely associated with an increase of nuclear raptor. High cytoplasmic raptor expression indicated worse prognosis on long-term follow-up.

**Conclusion:**

We present a connection between raptor localization to the nucleus and ERα-positive breast cancer, suggesting raptor as a player in stimulating the growth of the luminal A subtype and a possible target along with endocrine treatment.

**Electronic supplementary material:**

The online version of this article (10.1007/s10549-017-4508-x) contains supplementary material, which is available to authorized users.

## Background

The majority of breast tumors are dependent on estrogen signaling for proliferation and survival. Approximately 25% of endocrine-treated breast cancers develop resistance during the course of treatment [1]. The phosphatidylinositol 3-kinase/Akt/mechanistic target of rapamycin (PI3K/Akt/mTOR) signaling pathway is a master regulator of cell growth and proliferation as a result of nutrient and growth factor availability [2] and is thought to contribute to endocrine therapy resistance, as hyperactivation of this pathway makes growth of tumors less hormone dependent [3].

Activation of mTOR signaling promotes cellular biosynthesis, proliferation, and accelerated cell aging by increasing senescence and reducing the reservoir of stem cells. Therefore, long-term inhibition of mTOR signaling may reduce the growth of tumors dependent on mTOR. However, systemic effects of mTOR inhibition include serious side effects, such as immune suppression, increase of blood glucose levels, and infertility [4, 5].

mTOR, in association with other proteins, forms two distinct complexes: mTOR complex 1 (mTORC1) and mTOR complex 2 (mTORC2). Raptor is an adaptor protein that allows the mTORC1 complex to bind and phosphorylate downstream targets such as the eukaryotic initiation factor 4E-binding protein 1 (4E-BP1) and the p70 ribosomal S6 kinase 1 (S6K1) [6–9]. In mice, embryonic knockout of either mTOR or raptor produced similar phenotype of embryonic arrest, indicating that raptor is essential for mTORC1 function [10, 11].

Aberrations in various upstream regulators of the mTOR signaling pathway leading to its upregulation are frequently noted in cancers, providing rationale for inhibition of the mTORC1 signaling pathway. mTOR inhibitors such as rapamycin and its analogs temsirolimus and everolimus are clinically approved treatments for several types of cancers [12]. Specifically, for ERα-positive breast cancer patients recurring on endocrine therapy, everolimus has been shown to prolong time to progression [13]. Further understanding of the mTORC1 signaling pathway and its contribution to breast cancer biology should facilitate the development of improved breast cancer treatment.

Importantly, a significant degree of cross-regulation exists between ERα and mTORC1 signaling pathways whereby mTORC1 activates ERα’s transcriptional activity, while estrogen activates mTORC1 [14–17]. We have recently discovered that ERα and raptor directly bind each other. ERα, upon estrogen stimulation, translocates raptor to the nucleus where mTORC1 activates the transcription of ERα target genes [18], indicating a role of raptor in ERα-positive breast cancer progression. Additionally, in vitro tamoxifen treatment of breast cancer stem cells resulted in endocrine resistance that could be reversed with an mTOR inhibitor [19].

Here we aimed to investigate whether there is a correlation between subcellular expression of raptor and ERα status of breast tumors. We set out to analyze raptor protein expression and localization in a randomized retrospective cohort of postmenopausal breast cancer patients [20]. We found that nuclear expression of raptor in luminal A-like breast tumors predicted a group of patients with good prognosis but with no clear benefit of tamoxifen treatment. Additionally, we found co-localization of raptor and ERα upon estrogen stimulation in ERα-positive, but not in ERα-negative breast cancer cells.

## Materials and methods

### Study cohort

During 1976–1990, a randomized trial with postmenopausal breast cancer patients was initiated investigating tamoxifen treatment compared with no endocrine treatment [20]. In the present analysis, the low-risk group not receiving chemotherapy was included. This design makes the cohort with collected primary tumors and long-term follow-up data unique, as a treatment predictive value of biomarkers can be assessed when comparing treated with non-treated patient groups [21, 22].

Paraffin-embedded tissues of 912 tumors were used in this study. Tumor tissue was collected on surgical removal of the primary tumor and incubated in formalin for fixation and paraffin embedded. Three cores of abundant tumor cell content were selected to represent each tumor on a tissue microarray (TMA). The ERα status was assessed as previously described [23]. For all proteins detected, a portion of samples was missing. In the supplementary table of a previously published paper, missing samples were compared with the samples on TMA and with samples of the original cohort [24]. The results show no bias in the missing cases with respect to tumor size, ERα status, or tamoxifen treatment. The present study was designed and presented with regard to the reporting recommendations for tumor marker prognostic studies (REMARK) guidelines [25].

### Protein detection

Specific protein content of the tumor cells was determined by immunohistochemistry. The PT Link station was used for deparaffinization and antigen retrieval in a low-pH buffer, starting at 65, 96 °C for 20 min and cooled down to 65 °C (DakoCytomation, Glostrup, Denmark). Inactivation of endogenous peroxidase in 3% hydrogen peroxide in water was followed by blocking in serum-free protein block for 10 min (Spring Bioscience, Freemont, CA). TMAs were incubated in a moisturized chamber at 4 °C overnight with the raptor antibody diluted 1:50 (EP539Y-ab40768, Abcam, Cambridge, UK). Secondary rabbit antibody was applied for 30 min, and protein was developed with DAB+chromogen (DakoCytomation) and counterstained with hematoxylin. All wash steps were in phosphate buffer saline including 0.5% bovine serum albumin. The tissue was dehydrated and cover glass was mounted with Pertex (Histolab).

### Antibody validation

The breast cancer cell line MDA-MB-231 was transfected with RPTOR siRNA. Protein detection with Western blot showed a specific band at 150 kDa that disappeared after knock-down (Supplementary Fig. A). In addition, a reduction of the direct downstream protein p-4EBP1-s65 was observed. Cells were formalin fixed and paraffin embedded to test the antibody’s specificity in the immunocytochemical setting. In cells treated with a control siRNA, raptor was highly expressed in the cytoplasm, whereas in the RPTOR siRNA-treated cells the expression was lower (Supplementary Fig. B).

### Scoring

**Fig. 1:**
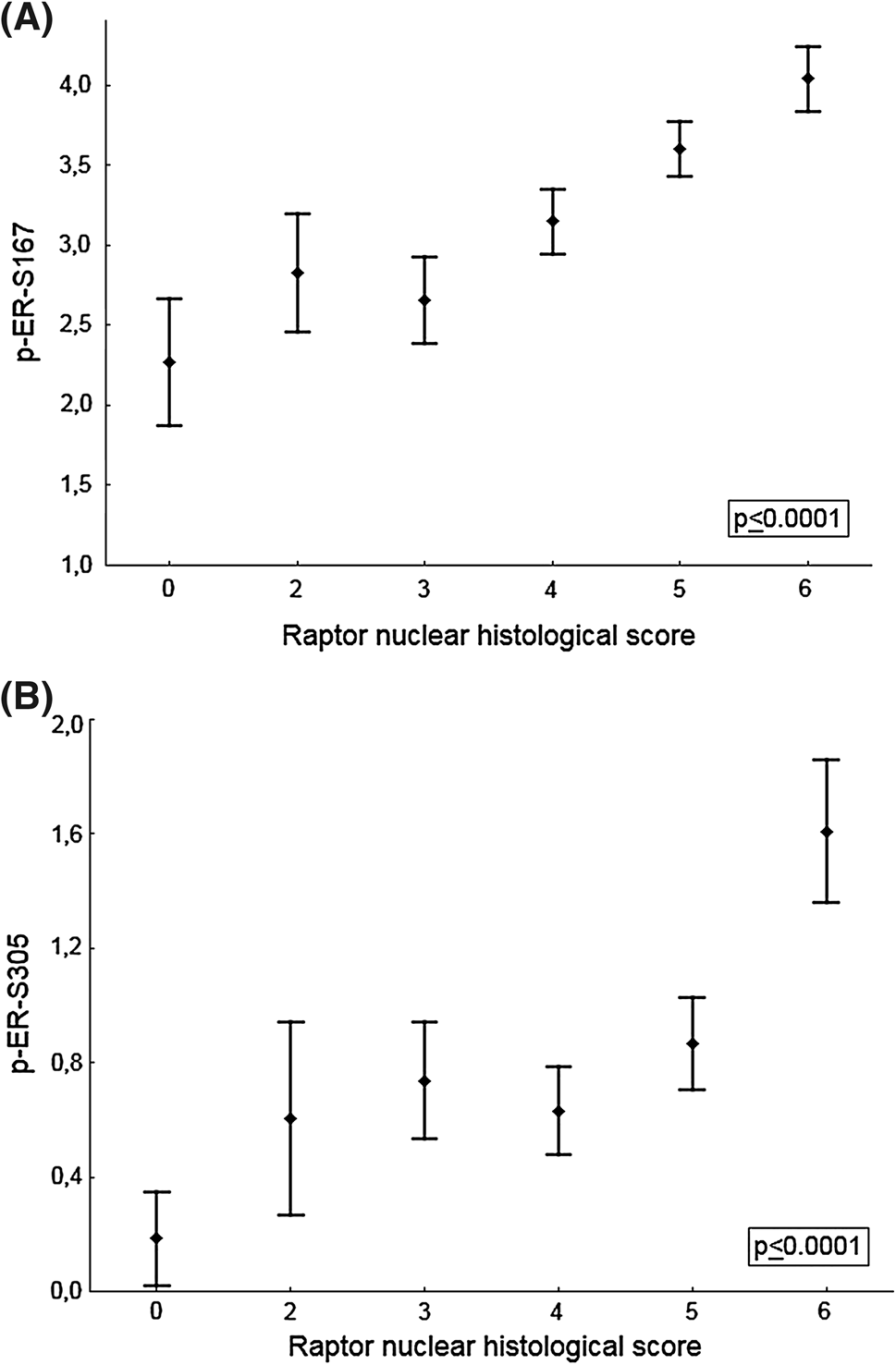
Raptor nuclear histological score (0, 2–6) correlated strongly with ER phosphorylations pER-S167 (**a**) and pER-S305 (**b**). Mean and 95% CI (confidence interval) plots of *p*-ER index grouped by raptor nuclear score. The *p* values refer to the Kruskal–Wallis H test

Raptor expression was evaluated on three separate core biopsies for each tumor. Protein expression in tumor cells was scored by two independent observers. For the cytoplasm, four steps of intensity were scored: negative, weak, medium, and strong. For further statistical analysis, negative and weak were considered as low, and medium and strong were considered as high. For the nucleus, four steps of intensity, negative, weak, medium, and strong, were evaluated along with four steps of frequency: 0 was <1%, score 1 was 1–25%, score 2 was 26–75%, and score 3 was >75%. A histological score was calculated by adding intensity to percentage score, with a final score of 0, 2–6, used in Fig. 1. For further analysis of nuclear raptor, to avoid groups with too small number of patients, the cases were divided into three as equally large groups as possible: low was score 0–3, medium was score 4, and high was score 5–6. Scoring information of other variables used in this study was previously published [24, 26].

### Cell culture and treatment

MCF7, ZR75-1, T47D, MDA-MB-231, MDA-MB-436, and MDA-MB-468 cells were obtained from American type culture collection (ATCC). Tamoxifen-resistant cells (Tam^R^) were generated by continuous culture of MCF7 cells in the presence of 100 nM 4-hydroxy-tamoxifen for 3 months. Cells were cultured in a humidified incubator with 5% CO_2_ at 37 °C in Dulbecco’s modified Eagle’s medium (DMEM) with 10% fetal bovine serum (FBS), 100 I.U. Penicillin, and 100 µg/mL Streptomycin. For experiments, the cells were grown in phenol red-free media with 10% charcoal-stripped FBS for 3 days. All cells were starved in serum-free media for 24 h and stimulated with 10 nM estradiol for 30 min.

### Fractionation assay

Following treatment, MCF7, MCF7-Tam^R^, ZR75-1, T47D, MDA-MB-231, and MDA-MB-436 cells were harvested and cytoplasmic and nuclear fractionation was performed using NE-PER^®^ Nuclear and Cytoplasmic Extraction Reagent kit (ThermoFisher Scientific, Hampton, NH) according to the manufacturer’s instructions. Samples were subsequently denatured using LDS Sample buffer and Reducing agent (Invitrogen, Carlsbad, CA) at 70 °C for 10 min. Samples were resolved using Bis–Tris Plus gels (Invitrogen, Carlsbad, CA) and transferred onto nitrocellulose membrane (GE Healthcare, Port Washington, NY). Membranes were probed with the following primary antibodies: ERα (sc8005), raptor (sc81537), and mTOR (sc-1549) (Santa Cruz Biotechnology, Dallas, TX), p-ERK (M8159) Sigma-Aldrich (St Louis, MO), PARP (Abcam, Cambridge, UK), p-mTOR-S2448 (5536S), p-Akt-S473 (4060L), Akt (4691X), p-PRAS40-T246 (13175P), p-S6K-T389 (9206S), 4EBP1 (9644S), p-4EBP1-S65 (9454S), and ERK (4695) (Cell Signaling Technologies (Danvers, MA). Signal detection and quantification were accomplished using IRDye-conjugated anti-rabbit (LI-COR, 827-08365, Lincoln, NE), anti-mouse (LI-COR, 926-68070, Lincoln, NE), or anti-goat (LI-COR, 926-68074, Lincoln, NE) secondary antibodies using Odyssey infrared detection instrument (LI-COR, Lincoln, NE). All immunoblots were performed at least thrice to ensure reproducibility.

### Immunofluorescence

MCF7 cells were plated on poly-l-lysine-coated cover slips (Fisher, Hampton, NH), while T47D, MDA-MB-231, and MDA-MB-468 cells were plated on Geltrex^®^-coated cover slips (Invitrogen, Carlsbad, CA). Following treatment, the cells were fixed in 1% PFA for 10 min, washed twice with PBS, subsequently permeabilized in 0.3% NP-40/PBS for 10 min, and blocked in Image-iT FX signal enhancer solution (Invitrogen, Carlsbad, CA) for 30 min. Cells were incubated with ERα (1:50 dilution, SC-8005 Santa Cruz Biotechnology, Dallas, TX) and raptor (1:400, ab169506 Abcam, Cambridge, UK) primary antibodies in 1% BSA/PBS overnight at 4 °C. Cover slips were subsequently washed in PBS and incubated with Alexa Fluor 488 goat anti-mouse and Alexa Fluor 555 goat anti-rabbit secondary antibodies (1:500 dilution, Invitrogen, Carlsbad, CA) for 1 h at room temperature in the dark. Following 5-min incubation with DAPI, cover slips were mounted using an Image-iT^®^ FX signal enhancer (Invitrogen, Carlsbad, CA) and imaged using a Nikon fluorescent microscope under ×40 magnification.

### Statistical methodology

Statistical analyses were performed using Statistica 12 (StatSoft/Dell Software, TULSA, OK). For comparisons of raptor protein expression with prognostic and clinical characteristics, the Pearson *χ*^2^ test was applied for 2 × 2 tables. For rank correlation, the Spearman rank order correlation test was applied. Correlation of raptor nuclear histological score *vs* ERα phosphorylation levels was done with the Kruskal–Wallis H test. Relative risks of distant metastasis were estimated using the Cox proportional hazards model. Distant metastasis-free survival (DMFS) time distributions were compared with the log-rank test and plots were drawn with the Kaplan–Meier method, visualizing time from randomization to first event of distant metastasis. Cox proportional hazards regression was used in interaction analysis exploring raptor expression as a potential predictive factor of tamoxifen benefit. A *p* value <0.05 was considered significant, with the exception of Table 1 where a *p* value <0.01 was considered significant due to adjustment of multiple comparisons.

**Table 1:** Correlations between raptor protein expression in the cytoplasm, intensity low and high, and in the nucleus, three graded scores of intensity and percentage positive nuclei low, medium, and high, with clinical and pathological variables

	Raptor in the cytoplasm			Raptor in the nucleus intensity score			
	Low	High	*p* (*r*_s_)	Low	Medium	High	*p* (*r*_s_)
All	368 (49)	388 (51)		238 (31)	192 (25)	326 (43)	
No tamoxifen	181 (49)	187 (51)		109 (30)	103 (28)	156 (42)	
Tamoxifen	187 (48)	201 (52)	0.079 (–)	129 (33)	89 (23)	170 (44)	0.78 (–)
Size <20 mm	284 (50)	284 (50)		164 (29)	131 (23)	273 (48)	
Size >20 mm	75 (44)	95 (56)	0.18 (–)	70 (41)	53 (31)	47 (28)	**0.00001 (−0.16)**
NHG 1	66 (55)	54 (45)		25 (21)	28 (23)	67 (56)	
NHG 2	183 (49)	194 (51)		112 (30)	87 (23)	178 (47)	
NHG 3	61 (42)	85 (58)	0.031 (–)	66 (45)	47 (32)	33 (21)	**<0.00001 (−0.22)**
ER negative	70 (42)	95 (58)		73 (44)	51 (31)	41 (25)	
ER positive	288 (51)	281 (49)	0.064 (–)	162 (28)	132 (23)	275 (48)	<*0.00001 (0.18)*
PgR negative	152 (47)	173 (53)		132 (41)	79 (24)	114 (35)	
PgR positive	175 (51)	169 (49)	0.29 (–)	86 (25)	80 (23)	178 (52)	<*0.00001 (0.19)*
HER2 negative	312 (51)	304 (49)		192 (31)	153 (25)	271 (44)	
HER2 positive	30 (36)	54 (64)	0.010 (–)	34 (40)	24 (29)	26 (31)	0.024 (–)
pAKTs473 c low	174 (62)	108 (38)		115 (41)	67 (24)	100 (35)	
pAKTs473 c high	179 (40)	271 (60)	<*0.00001 (0.21)*	115 (26)	119 (26)	216 (48)	*0.00002 (0.16)*
pAKTs473 n low	163 (52)	148 (48)		162 (52)	81 (26)	68 (22)	
pAKTs473 *n* high	190 (45)	231 (55)	0.051 (–)	68 (16)	105 (25)	248 (59)	<*0.00001 (0.42)*
p-mTORs2448 low	309 (48)	330 (52)		210 (33)	166 (26)	263 (41)	
p-mTORs2448 high	41 (45)	50 (55)	0.056 (–)	19 (21)	20 (22)	52 (57)	*0.0032 (0.11)*
pS6Kt389 c low	178 (58)	131 (42)		101 (33)	82 (27)	126 (41)	
pS6Kt389 c high	174 (41)	251 (59)	*0.00001 (0.16)*	128 (30)	105 (25)	192 (45)	0.27 (–)
pS6Kt389 n low	229 (51)	222 (49)		192 (43)	121 (27)	138 (31)	
pS6Kt389 n high	123 (44)	159 (56)	0.059 (–)	37 (13)	65 (23)	180 (64)	<*0.00001 (0.36)*
pERs167 low	292 (50)	296 (50)		214 (36)	157 (27)	217 (37)	
pERs167 high	73 (45)	90 (55)	0.27 (–)	23 (14)	34 (21)	106 (65)	<*0.00001 (0.24)*
pERs305 low	247 (52)	225 (48)		175 (37)	134 (28)	163 (35)	
pERs305 high	107 (41)	153 (59)	*0.0038 (0.11)*	55 (21)	53 (20)	152 (58)	<*0.00001 (0.23)*
S6K1 c low	335 (51)	325 (49)		207 (31)	171 (26)	282 (43)	
S6K1 c high	26 (31)	57 (69)	*0.00084 (0.12)*	25 (30)	20 (24)	38 (46)	0.66 (–)
S6K1 n low	311 (50)	307 (50)		203 (33)	162 (26)	253 (41)	
S6K1 n high	50 (40)	75 (60)	0.035 (–)	29 (23)	29 (23)	67 (54)	*0.0072 (0.10)*
Cyclin D1 low	240 (53)	215 (47)		165 (36)	125 (27)	165 (36)	
Cyclin D1 high	121 (44)	152 (56)	0.028 (–)	65 (24)	64 (23)	144 (53)	*0.00001 (0.17)*

Pearson χ^2^ analysis was used for 2 × 2 correlations, and Spearman rank order analysis was used for 3 × 2 and 3 × 3 variable correlations. A *p* value <0.01 was considered significant. Spearman rank order correlation values (*r*_s_) are given in case of significance. Bold *p* values indicate a significant negative correlation and italic *p* values indicate a significant positive correlation

## Results

### High nuclear raptor expression in luminal A-like tumors

Consistent with our previously published data, we identified an association between high nuclear raptor expression and the luminal A features, namely ERα positive, HER2 negative, and low Nottingham histological grade (NHG) (Table 1). We found statistical correlations between nuclear raptor localization and phosphorylation of both ERα and mTORC1 signaling pathway components (Table 1), indicating a relationship between raptor and mTORC1-driven phosphorylation of ERα.

To confirm the specificity of our findings, we looked at the correlation between subcellular raptor localization and cyclin D1 expression, an ERα-regulated gene and a common surrogate for measuring ER transcriptional activity. Indeed, we observed a significant correlation between high levels of cyclin D1 expression and nuclear raptor localization (Table 1). Furthermore, the correlation of nuclear raptor expression with two other ERα-regulated genes, PgR and S6K1, further strengthens our hypothesis that nuclear raptor expression induces the transcription of ERα-regulated genes. The correlations of nuclear raptor with PgR and cyclin D1 were still significant, whereas cytoplasmic p-mTOR and nuclear S6K1 only showed trends of association to raptor in an analysis restricted to ERα-positive tumors (data not shown).

Additionally, we identified a statistically significant correlation between the cytoplasmic localization of raptor and cytoplasmic phosphorylation of S6K1 on T389 and Akt on S473, indicating that raptor also activates mTORC1 signaling in the cytoplasm, as expected.

### Relationship between raptor and ER phosphorylated at serine 167 or 305

Recently, we demonstrated that mTOR directly phosphorylates ERα on S104/106 as a result of raptor binding to the TOS motif (591-FPATV-595), in the C terminus of ERα [18]. Analysis of the expression levels of ERα phosphorylated on S167 and S305 in tumors showed statistically significant increased nuclear raptor expression as ERα becomes phosphorylated on S167 (*r*_s_ = 0.36, Fig. 1a) and S305 (*r*_s_ = 0.28, Fig. 1b). Importantly, work from our and other labs has shown a connection between the S305 phosphorylation and endocrine resistance [24, 27], although the endocrine response in relation to S167 phosphorylation was not as clear-cut [28, 29].

### Induced tamoxifen resistance caused an increase of nuclear raptor translocation

**Fig. 2:**
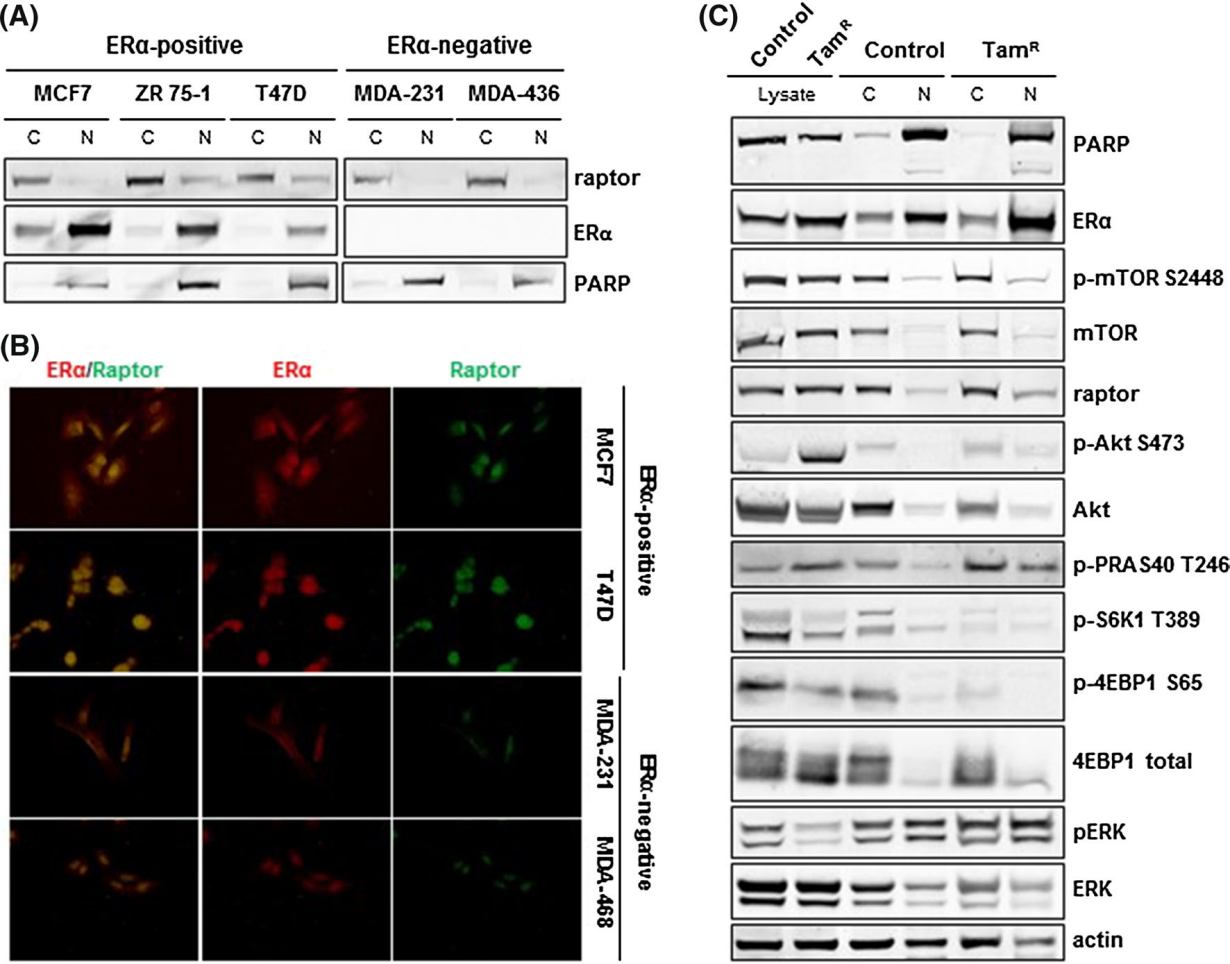
Raptor expression in ERα-positive, ERα-negative, and tamoxifen-resistant breast cancer cells. Raptor expression was higher in ERα-positive than in ERα-negative cell lines by Western blot analysis (**a**). Comparing ERα-positive with ERα-negative cells shows raptor in the ERα-positive but not in the ERα-negative nuclei as observed by in situ immunofluorescence (**b**). Raptor presence increased in the nucleus (N) in MCF7 tamoxifen-resistant cells (Tam^R^) compared with MCF7 parental cells (Control), and changes in mTORC1 and Akt signaling were observed (**c**)

To further investigate nuclear function of raptor, we analyzed subcellular localization of raptor in ERα-positive and ERα-negative breast cancer cells (Fig. 2a). In ERα-positive MCF7, ZR-75-1, and T47D cells, raptor was present in the nuclear fraction, whereas it was absent from the nucleus of ERα-negative MDA-MB-231 and MDA-MB-436 cells. The association between nuclear ERα expression and raptor was also visualized in situ by immunofluorescence in MCF7, T47D, MDA-MB-231, and MDA-MB-468 cells (Fig. 2b), suggesting a close relationship between ERα and raptor in the nucleus.

To study the effects of tamoxifen treatment of ERα-positive breast cancer cells, ERα-positive MCF7 cells were treated with tamoxifen for 3 months to achieve tamoxifen resistance. Analysis of subcellular nuclear and cytoplasmic fractions of control as well as tamoxifen-resistant (Tam^R^) MCF7 cells showed that mTOR and raptor were still observed in the nucleus of tamoxifen-resistant cells and the ERα levels were upregulated (Fig. 2c). Further, we detected an increase in phosphorylation of both AKT on S473 as well as its downstream target PRAS40 on T246 in tamoxifen-resistant cells. This was accompanied by the downregulation of total p-S6K1 on T389 and p-4EBP1 on S65, markers of downregulation of mTORC1 signaling; however, changes in the relative distribution between cytoplasmic and nuclear expression of the proteins were unclear. Compared to the changes observed in PI3K/mTOR signaling, long-term tamoxifen treatment did not affect the subcellular distribution of ERK, a marker of the MAPK signaling pathway. Although phosphorylated ERK levels decreased in whole cell lysates, its cytoplasmic and nuclear distribution remained unchanged. Nuclear expression of PARP served as an internal control.

### The reduction of distant recurrence rate with tamoxifen is related to nuclear raptor expression

**Fig. 3:**
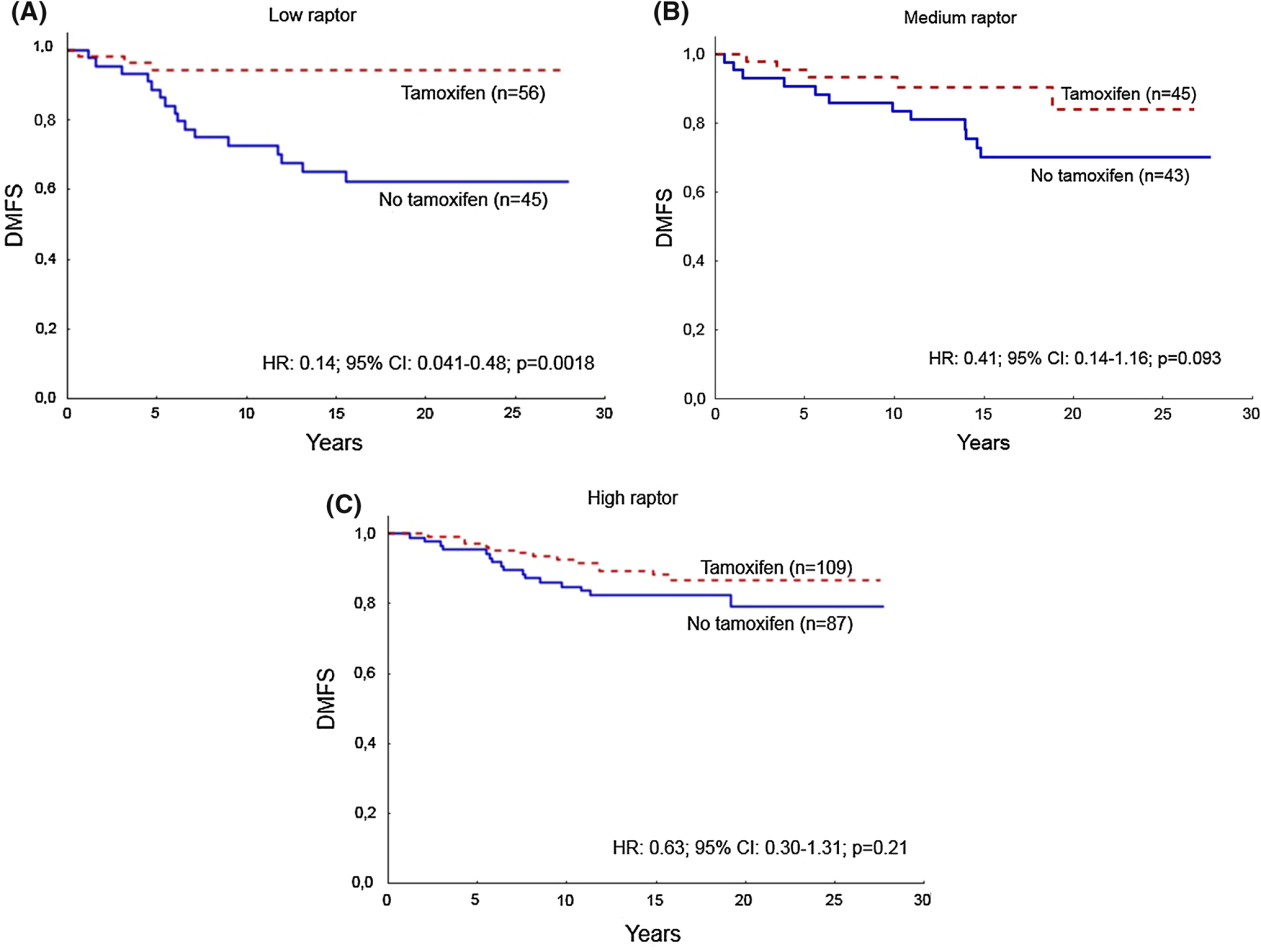
Tamoxifen response is inversely associated with increased nuclear raptor score. Tamoxifen is beneficial for patients with low score of nuclear raptor in luminal A-like graded tumors (**a**). The tamoxifen response is sequentially reduced with increased score of nuclear raptor, showing a trend toward benefit in the group of medium score (**b**), and no significant benefit in the group of high score (**c**). The interaction between tamoxifen response and raptor showed a significant decrease in benefit with higher score (*p* = 0.036). Distant metastasis-free survival (DMFS)

Due to the newly identified link between ERα and raptor [18], we examined the patient benefit from tamoxifen in relation to raptor expression and its subcellular localization in luminal A-like tumor. Analysis of luminal A-like tumors based on the levels of nuclear raptor with respect to tamoxifen treatment showed that low nuclear raptor expression correlated with beneficial response to tamoxifen treatment and significantly decreased risk of distant recurrence (hazard ratio (HR) 0.14; 95% confidence interval (CI) 0.041–0.48; *p* = 0.0018) (Fig. 3a). Moreover, medium-level raptor expression in the nucleus trended toward benefit from tamoxifen treatment, but the risk was not significantly reduced (HR 0.41; 95% CI 0.14–1.16; *p* = 0.093) (Fig. 3b). For patients with tumors showing high nuclear raptor expression, the risk of distant metastases was not significantly reduced with tamoxifen treatment (HR 0.63; 95% CI 0.30–1.31; *p* = 0.21) (Fig. 3c). The difference in tamoxifen benefit between the three groups was significant, *p* = 0.036, indicating that higher levels of nuclear raptor expression are associated with less benefit from tamoxifen treatment.

### Raptor localization shows diverse prognosis

**Fig. 4:**
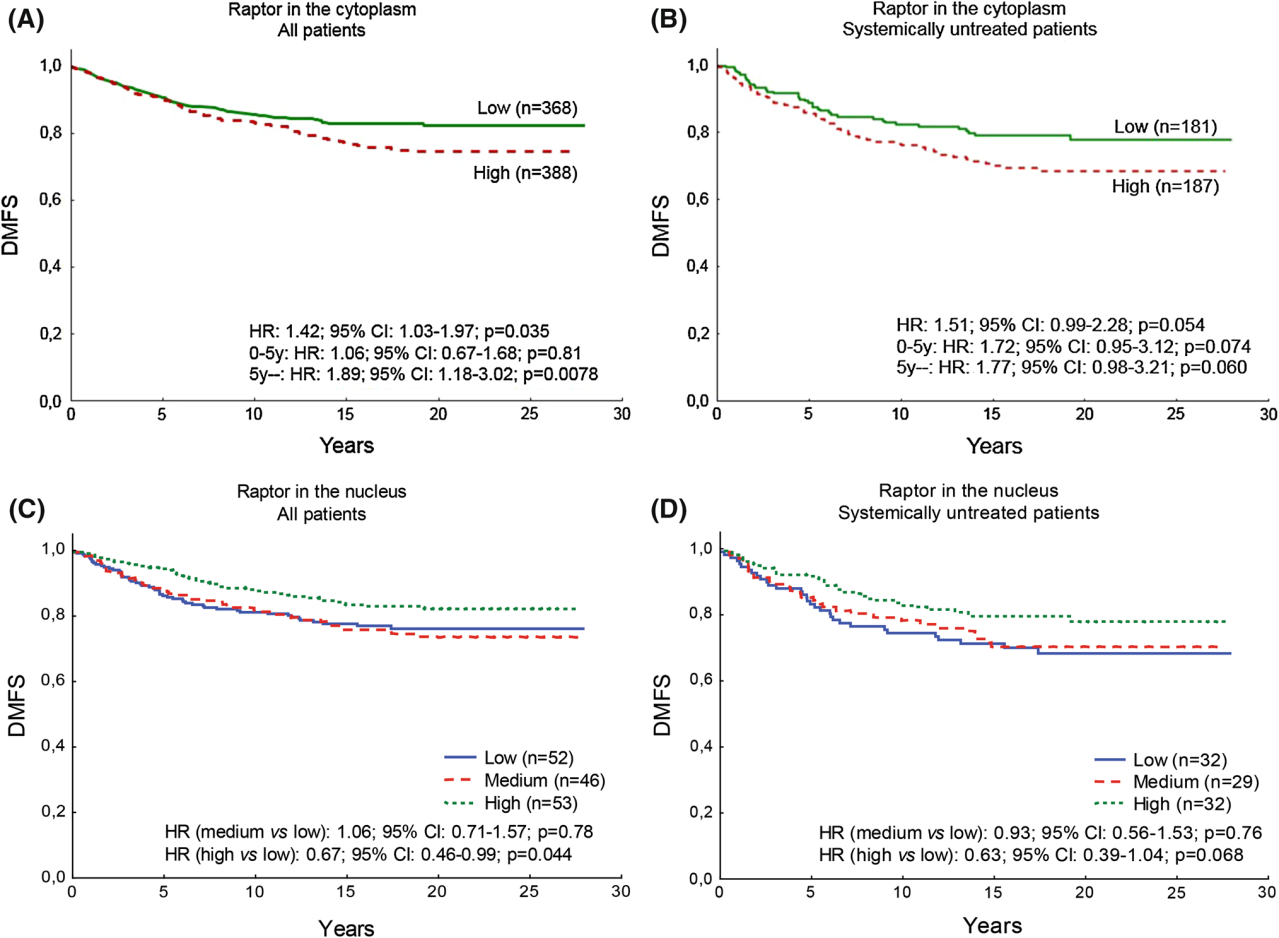
Raptor as a prognostic marker shows diverse outcomes depending on localization. **a** High intensity of raptor in the cytoplasm indicated a significantly worse prognosis. This was not evident during the first 5 years after randomization. For the group of patients that had no distant recurrences within the first 5 years, the raptor intensity in the cytoplasm had significant impact on the distant metastasis rate. All patients were included in the analysis. **b** High intensity of raptor in the cytoplasm tended to be significantly worse for systemically untreated patients. No difference was observed for the time periods before and after 5 years from randomization. **c** Low, medium, and high scores of raptor in the nucleus of all patients, *p* = 0.031. **d** Low, medium, and high scores of raptor in the nucleus of systemically untreated patients, *p* = 0.13

A high cytoplasmic raptor expression indicated a worse prognosis (DMFS HR 1.42; 95% CI 1.03–1.97; *p* = 0.035) (Fig. 4a). During the first five years after diagnosis, raptor expression in the cytoplasm showed no prognostic value; however, after five years it had a strong prognostic impact with an 89% risk increase compared with patients with low cytoplasmic raptor in the tumor (DMFS HR 1.89; 95% CI 1.18–3.02; *p* = 0.0078). Analysis of the tamoxifen-untreated subgroup indicated a similar result as for all patients although not significant (HR 1.51; 95% CI 0.99–2.28; *p* = 0.054) (Fig. 4b). In a multivariate analysis adjusting for treatment, tumor size, grade, ER, and HER2, the prognostic value of cytoplasmic raptor expression was observed to be significant when analyzing all patients (HR 1.43, 95% CI 1.00–2.06, *p* = 0.049), with a similar trend for systemically untreated patients (HR 1.47, 95% CI 0.93–2.33, *p* = 0.098).

In contrast to what was found for cytoplasmic raptor, high expression of nuclear raptor indicated an improved prognosis for breast cancer patients when the group with low expression was compared with the group with high expression (DMFS HR (medium vs low): 1.06; 95% CI 0.71–1.57; *p* = 0.78) and (DMFS HR (high vs low): 0.67; 95% CI 0.46–0.99; *p* = 0.044) (Fig. 4c). A similar trend was observed in the group of tamoxifen-untreated patients (DMFS HR (medium vs low): 0.93; 95% CI 0.56–1.53; *p* = 0.76) and (DMFS HR (high vs low): 0.63; 95% CI 0.39–1.04; *p* = 0.068) (Fig. 4d). Nuclear raptor expression did not show significance for prognosis in the multivariate analysis.

## Discussion

The previous experimental study has demonstrated that when stimulated with estrogen, ERα recruits mTORC1 into the nucleus, which increased ERα transcriptional activity [18]. In the current study, we show that our results are in line with these findings in clinical material and associations between nuclear raptor localization, low tumor grade, and ERα/PgR status of breast tumors. A previous study of a small breast cancer cohort found raptor mRNA expression to correlate with higher tumor grade [30]. In that study, mRNA was extracted from whole cell lysates, which is more representative of raptor expression in the cytoplasm rather than that of the nucleus, which may explain the apparent difference with our observations.

The ERα and PI3K/mTORC1 signaling pathways regulate cell growth and survival and are important for breast tumor development as these pathways are often dysregulated in breast cancer. Due to their cross-regulation, it is difficult to inhibit either one of them as inhibition of one of the pathways can result in the upregulation of signaling from the other pathway [31]. ERα can be activated by either its ligand, 17β-estradiol (E2), or through growth factors by phosphorylation at specific residues [32], and we have previously analyzed protein expression of phosphorylated ERα at S167 and S305 [26]. In the current work, we showed the correlations between nuclear raptor localization and phosphorylation of both ERα and mTORC1 signaling pathway components, indicating a relationship between raptor and mTOR-driven phosphorylation of ERα. The biological significance of the associations presented in Table 1 needs to be considered carefully as strong statistical significance is easily achieved when the number of observations is high. However, also when considering the r-values some of the correlations are relatively strong with *r*_s_ = 0.36 for the correlation between raptor and pERs167 (Fig. 1a) and considering the associations of raptor with pAKTs473 and pS6Kt389 (*r*_s_ = 0.42 and *r*_s_ = 0.36, respectively). This is in line with our previous observation of a mechanism that appears to be two-pronged, whereby raptor, binding to the TOS motif of ERα, facilitates direct phosphorylation by mTOR on S104/106 and mTOR-activated kinase S6K1 phosphorylates ERα on S167 of the activation function 1 domain [15, 18, 33], promoting ERα activation.

Therefore, in estrogen-dependent tumors, the mTORC1 signaling pathway activates ERα signaling to stimulate tumor growth. When the estrogen levels are decreased by aromatase inhibitors or the estrogen action is counteracted by tamoxifen, the mTORC1 pathway could potentially maintain ERα activity at a lower but still steady level via a ligand-independent mechanism, leading to late relapses in spite of adjuvant therapy. Our findings may become clinically important as they indicate a subgroup of ERα-positive patients that benefit poorly from tamoxifen as well as implicate raptor as a potential target for inhibition in endocrine-dependent tumors.

Endocrine resistance may develop during tamoxifen treatment when some tumors switch from luminal A to luminal B subtype, with a loss of PgR expression. In the current study, we found raptor to be preferentially expressed in the nucleus of PgR-expressing tumors. Since only primary tumors were available for analysis, it would be interesting to investigate subcellular raptor localization in metastatic tumors with respect to tamoxifen response.

We observed that high cytoplasmic raptor expression indicated a worse prognosis, a finding that was consistent with the role of increased oncogenic cytoplasmic mTORC1 signaling in breast cancer. Knock-down of either raptor or rictor mitigated the effect of radiation-induced apoptosis, by decreasing entry into S-phase and inducing cell cycle arrest in both G1 and G2 phases [34]. This is in line with the proposed function of raptor as a general oncogenic protein as we observed cytoplasmic raptor association with poor breast cancer outcome.

Why raptor in the nucleus correlates with a less malignant phenotype is not fully understood. When raptor enters the nucleus, this results in a concurrent reduction in cytoplasmic raptor levels and mTORC1 activity. If the tumor was dependent on mTORC1 in the cytoplasm for its continuous growth, raptor transfer to the nucleus could depress mTORC1-driven oncogenic activity. Further, nuclear raptor may represent indolent tumors growing under conditions of low levels of estradiol. We have noted the associations of nuclear raptor with the steroid-converting enzymes such as aromatase and 17β-HSD2 that could favor this view (unpublished data). We speculate that a low nuclear raptor expression is found in tumors with strong dependence on estradiol, the most potent ERα activator. Vice versa, high nuclear raptor expression is found in tumors less dependent on estradiol and more dependent on growth factor signaling, called as crosstalk.

## Conclusions

Raptor is a key component of mTORC1-driven signaling in breast cancer. We demonstrate that raptor localized in the cytoplasm of tumor cells, probably independent of ERα signaling, is an unfavorable prognostic sign. Interestingly, raptor presence in the nucleus seems to be involved in maintaining ERα-dependent growth despite endocrine treatment, probably representing indolent forms of luminal breast cancer. Accordingly, nuclear raptor expression was associated with good outcome, although with reduced clinical benefit from tamoxifen. Hence, we suggest a two-sided raptor function in close relation with estrogen signaling that can be visualized by subcellular localization: a cytoplasmic raptor with an ER-independent and a nuclear raptor with an ER-cooperative role.

## Electronic supplementary material

Below is the link to the electronic supplementary material.

Supplementary material 1 (DOCX 4558 kb)

## Abbreviations

ER: Estrogen receptor
PgR: Progesterone receptor
PI3K: Phosphatidylinositol 3-kinase mechanistic target of rapamycin
mTOR: Mechanistic target of rapamycin
mTORC1: mTOR complex 1
mTORC2: mTOR complex 2
raptor: Regulatory-associated protein of mTOR
4E-BP1: Eukaryotic initiation factor 4E-binding protein 1
S6K1: p70 Ribosomal S6 kinase 1
DMSF: Distant metastasis-free survival
IHC: Immunohistochemistry
TMA: Tissue microarray
FBS: Fetal bovine serum
NHG: Nottingham histological grade
Tam^R^: Tamoxifen-resistant MCF7 cells
HR: Hazard ratio
CI: Confidence interval
