# Supplementary material for: Raptor localization predicts prognosis and tamoxifen response in estrogen receptor-positive breast cancer
Source: Breast Cancer Res Treat. 2017 Nov 11;168(1):17–27. doi: 10.1007/s10549-017-4508-x (PMC5847064; doi:10.1007/s10549-017-4508-x)

**Supplementary figures**

**Antibody validation** Raptor was detected with Western blot in MDA-MB-231 breast cancer cells transfected with control siRNA and was removed with RPTOR siRNA. The downstream phosphorylated 4EBP1 at serine 65 was downregulated when raptor was knocked-down. GAPDH was used as loading control (**A**). Cells treated with siRNA were formalin fixed and paraffin embedded and analyzed with raptor antibody with immunocytochemistry. Raptor was detected in the cytoplasm and in the nucleus of control siRNA-transfected cells and markedly reduced in both compartments upon RPTOR siRNA transfection (**B**).


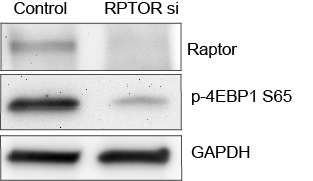


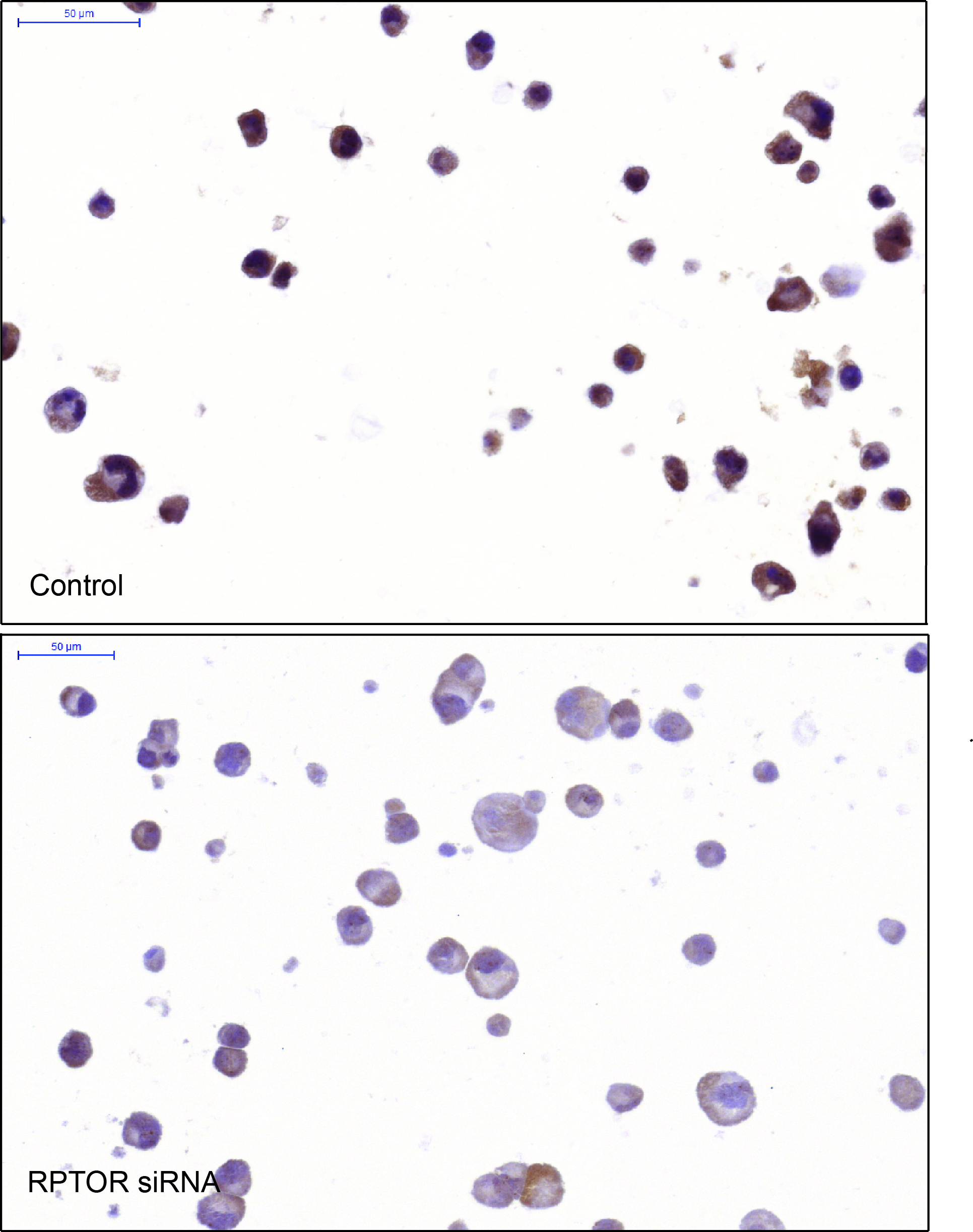

Supplement: Supplementary file 1 — Supplementary material 1 (DOCX 4558 kb) [file 10549_2017_4508_MOESM1_ESM.docx]
